# Supplementary material for: Tunneling of Mesh during Ventral Rectopexy: Technical Aspects and Long-Term Functional Results
Source: J Clin Med. 2022 Dec 30;12(1):294. doi: 10.3390/jcm12010294 (PMC9821569; doi:10.3390/jcm12010294)
Supplement: Supplementary file 1 [file jcm-12-00294-s001.zip › jcm-1998055-supplementary.pdf]

Supplementary Material

Table S1. Patients' distribution between standard and modified ventral rectopexy in open and minimally invasive surgery.

	**Open surgery [since 2010] n°(%)**	**Minimally invasive surgery [since 2018] n°(%)**
**Patients**	97	20
**Standard ventral rectopexy**	42 (43.3)	10 (50.0)
**Modified ventral rectopexy**	55 (56.7)	10 (50.0)
**Conversion from modified to standard ventral rectopexy**	4 (4.1)	2 (10.0)
